# Surgical Cases

**Published:** 1857-06

**Authors:** J. C. Hughes

**Affiliations:** Professor of Surgery in the Medical Department of the Iowa State University


### SURGICAL CASES. No. 1.

REPORTED BY J. C. HUGHES, M. D.

Professor of Surgery in the Medical Department of the Iowa State University.

Adolphe Dagenhardt—age 30—a German by birth, but for several years a resident of this State—had for the last two or three years suffered from disease of the urinary organs. He consulted a number of physicians in relation to his case, and placed himself under the treatment of as many. Some of them having examined him, diagnosed stone in the bladder, but Dr. E——, who had charge of the case for some time, was not satisfied with regard to the presence of stone, and placed him under treatment for what he considered chronic inflammation of the walls of that viscus and the kidneys.

Although improving slowly under the Doctor's treatment he was not satisfied, (believing his disease to be gravel,) and placed himself under the care of Dr. C——, of Illinois, who, after examining him, diagnosed stone in the bladder, and after the introduction of the sound was more fully confirmed of the correctness of his diagnosis. The Dr. not wishing to treat the case, wrote me upon the subject, asking me to operate. I immediately responded, requesting the patient to be brought to this city where I could have charge of the case.

The Dr. accompanied the case in May last, and on his arrival, desired me to operate at the earliest moment possible, it being necessary for him to return immediately. I objected to fixing a time, not yet having examined the case, but after being again assured by the Dr. that the stone was present, and that his condition was such as to justify an operation immediately, I consented to the hour of 11 o'clock next day, and after having invited several gentlemen of the profession to witness the operation, proceeded to the Hospital, accompanied by the Dr. to examine the case.

Now for the symptoms. The patient had not suffered from the journey, (which was by boat,) and the symptoms present were those of every day occurrence. Tongue and skin almost natural—pulse a little below the natural standard in volume—bowels regular—pain in the hypo-gastric and lumbar regions—incontinence of urine, accompanied by great pain, which subsided after the bladder was emptied. The urine passed in a small stream and frequently only guttatim—always pale and fully charged with muco-purulent matter—sometimes tinged with blood, and leaving a large deposite after a few hours standing—a great desire to pull the prepuce and glans penis, keeping the organ almost continually in his hand. I proceeded to introduce the sound, and at a short distance within the meatus urinarius I encountered stricture of the urethra, but not so much as to prevent the sound from passing, and, upon entering the membranous portion, found it impossible to pass the sound. I withdrew the instrument and substituted one of a smaller size, which, with difficulty I entered, its presence producing great suffering, and it was only after the administration of chloroform that I was enabled to make a satisfactory examination. Upon changing the position of the sound within the viscus, I found it contracted, thickened, and extremely dense, resembling to the touch, almost the click attendant upon the presence of stone. Yet I could discover no *calculus* and indicated the fact to the Dr. who at once remarked that if I would give him the staff he would point it out to me. I did so, and bringing the sonnd in contact with the dense structure before mentioned, he insisted upon the presence of a foreign body.

Having extended the examination as far as was justifiable upon this oacasion without finding stone, the time appointed for the operation was postponed. After a subsequent examination with the Dr. I was still unable to concur with him in diagnosis, and we thought best to defer any farther examination, the Dr. returning home, and the patient being placed upon treatment with the hope of improving the condition of the bladder and relieving the stricture which was present. The patient slowly improved, and in the meantime I had Prof. Marsh analyze the urine which was found to contaion *Phosphate of Lime* in large quantity.

I made a third examination with no better success and wrote the Dr. to that effect. So sanguine was he of the correctness of his diagnosis, that he replied, stating that if I did not operate, he would remove the case to Chicago. This, however, was not done, and the case continued under my charge, with some improvement in symptoms from time to time, for a period of two months, but without permanency, when he was attacked with dysentery, and on the 30th of July, died.

Upon examination after death, the following pathological conditions presented themselves:

In the membranous portion of the urethra, a fibrous band was stretched across the canal, which formed the obstruction to the entrance of the large sized sound, the smaller one having passed on either side. This was the result of inflammation, but as no venerial taint of system now or ever had existed, (taking the patient's statement as true,) it must have been the result of inflammatory action established by the passage of the irritating contents of the bladder.

The bladder was so contracted and thickened, that its cavity would not contain more than one ounce of fluids; at some points, fissures extended into its substance which were filled with pus of an unhealthy character, and it was lined by a more or less perfectly organised pyogenic membrane. Other points presented elevations of cartilaginous hardness, and from these, when bringing the sound in contact, was produced that sensation so closely resembling the click of stone. The ureters were healthy, but the kidney of the left side was almost destroyed, the medullary portion entirely broken down, the cortical substance only remaining to form a sack for a quantity of unhealthy pus, while the urine was loaded with the phosphate of lime. Yet no calculus formation had taken place either in kidneys or bladder, which may be attributed to the want of a nucleus.

[TO BE CONTINUED.]

